# Identification of RNAIII-binding proteins in *Staphylococcus aureus* using tethered RNAs and streptavidin aptamers based pull-down assay

**DOI:** 10.1186/s12866-015-0435-3

**Published:** 2015-05-15

**Authors:** Xu Zhang, Qing Zhu, Tian Tian, Changlong Zhao, Jianye Zang, Ting Xue, Baolin Sun

**Affiliations:** School of Life Sciences, University of Science and Technology of China, Hefei, Anhui 230027 China; CAS Key Laboratory of Innate Immunity and Chronic Disease, University of Science and Technology of China, Hefei, Anhui 230027 China; School of Life Sciences, Anhui Agricultural University, Hefei, Anhui, 230036 China

**Keywords:** *Staphylococcus aureus*, RNAIII, tRSA, Pull-down assay, RNA-binding proteins

## Abstract

**Background:**

It has been widely recognized that small RNAs (sRNAs) play important roles in physiology and virulence control in bacteria. In *Staphylococcus aureus*, many sRNAs have been identified and some of them have been functionally studied. Since it is difficult to identify RNA-binding proteins (RBPs), very little has been known about the RBPs in *S. aureus*, especially those associated with sRNAs.

**Results:**

Here we adopted a tRNA scaffold streptavidin aptamer based pull-down assay to identify RBPs in *S. aureus*. The tethered RNA was successfully captured by the streptavidin magnetic beads, and proteins binding to RNAIII were isolated and analyzed by mass spectrometry. We have identified 81 proteins, and expressed heterologously 9 of them in *Escherichia coli*. The binding ability of the recombinant proteins with RNAIII was further analyzed by electrophoresis mobility shift assay, and the result indicates that proteins CshA, RNase J2, Era, Hu, WalR, Pyk, and FtsZ can bind to RNAIII.

**Conclusions:**

This study suggests that some proteins can bind to RNA III in *S. aureus,* and may be involved in RNA III function. And tRSA based pull-down assay is an effective method to search for RBPs in bacteria, which should facilitate the identification and functional study of RBPs in diverse bacterial species.

**Electronic supplementary material:**

The online version of this article (doi:10.1186/s12866-015-0435-3) contains supplementary material, which is available to authorized users.

## Background

*Staphylococcus aureus* is a human and animal pathogen that can cause multiple infectious diseases. The high infection ability of the bacterium depends on the production of many virulence factors, which are under control of multiple regulatory pathways. These regulators include transcriptional regulator proteins, two-component systems, and small RNAs (sRNAs).

sRNAs in bacteria range from 50 to several hundred nucleotides, and function through RNA-RNA base pairing to activate or block the translation, or to induce the degradation of target mRNA. RNAIII is a 514 nucleotides sRNA, and can regulate the expression of many virulence genes as well as some regulators at the post-transcriptional level. Binding between RNAIII and its targets can lead to translation activation, translation blocking, or mRNA degradation mediated by RNase III [1].

Nowadays, hundreds of sRNAs have been identified in *S. aureus*, but very little has been known about RNA-binding proteins (RBPs) involved in sRNA regulation in this bacterium. It has been widely recognized that Hfq acts as an important chaperone in many Gram-negative bacteria [2]. In *S. aureus*, Hfq can bind to specific short RNA [3, 4], but deletion of *hfq* has no significant impact on sRNA stability or regulation, suggesting that Hfq is not an RNA chaperone in *S. aureus* [5]. RNase III is the only ribonuclease proved to be important for sRNA regulation in *S. aureus*, and it degrades double-stranded RNA formed by base-pairing of RNAIII with its mRNA targets such as *spa*, *coa*, and *rot* [6]. sRNA regulation may involve some other ribonucleases, which have not been studied yet. Besides, a transcriptional regulator SarA may act as an RBP and affect mRNA stability [7]. Thus, it is appealing to illustrate the interaction between sRNA and their RBPs in *S. aureus* [1, 8].

RBPs are difficult to identify due to the lack of effective tags. In previous studies, RNA affinity chromatography was used to purify a c-myc binding protein [9]; biotin labeling was used for affinity purification of many RBPs [10–12]; and in recent years, some RNA aptamers have been developed to bind to specific molecules and can be used as affinity tags, including aptamers binding to MS2 protein [13], tobramycin [14], sephadex [15], streptomycin [16], and streptavidin [17]. To make the aptamer and bait RNA more stable, tRNA scaffold was developed [18], and then tRNA scaffold to a streptavidin aptamer (tRSA) was successfully invented as an affinity matrix, which can efficiently capture some transcript-specific RBPs from cell lysates [19].

Aiming to identify proteins binding to RNAIII, we carried out pull-down assay using tRSA as a tag in this study. The tethered RNAs were successfully captured by Streptavidin MagneSphere Paramagnetic Particles (SA-PMPs), and those proteins binding to RNAIII were isolated and analyzed by mass spectrometry (MS). Using this method, 81 proteins were identified, and RNA-binding abilities of 9 proteins were further determined by electrophoresis mobility shift assay (EMSA). Our data indicate that some proteins can bind to RNAIII, and that tRSA based pull-down assay is an effective method to identify RBPs in *S. aureus*. Further research on these RBPs should shed light on the function and mechanisms of sRNA regulation in *S. aureus*.

## Results

### tRSA-RNAIII and the RBPs were captured by SA-PMPs in the pull-down system

**Fig. 1:**
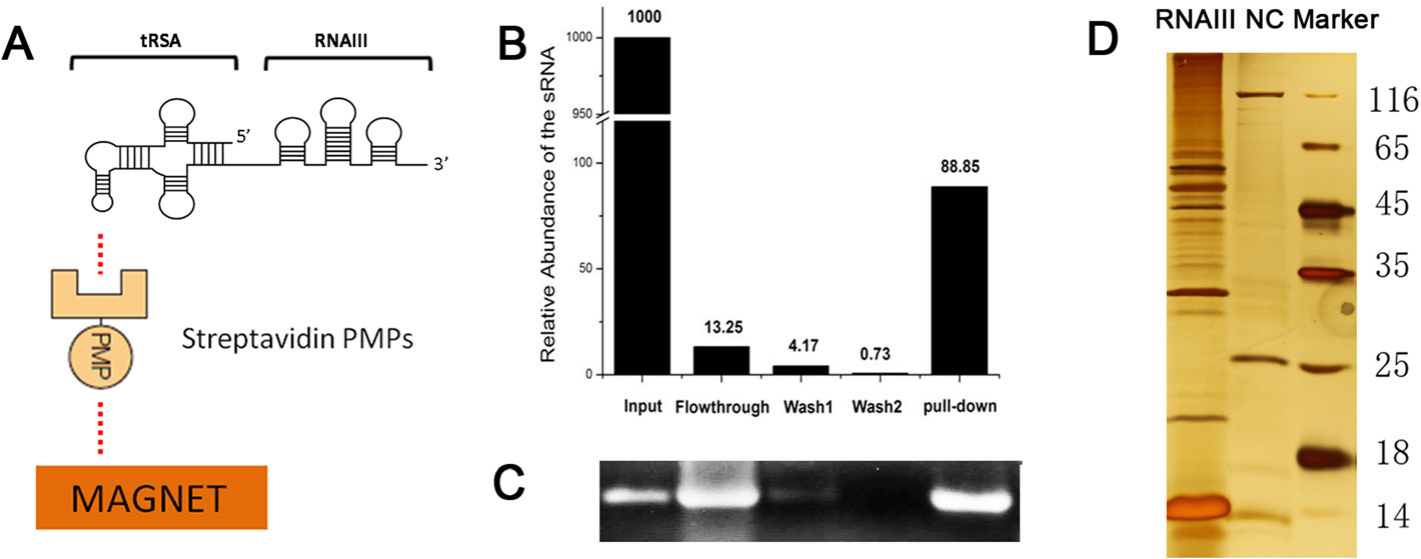
Procedure of the pull-down assay. **a** Composition of the pull-down system. RNAIII was attached to the 3′-end of the tRSA, and the SA-PMPs was used to capture the aptamer. **b** The tethered RNA was used for pull-down assay and the captured RNA in each step was collected and qualified by real-time RT-PCR. The RNA abundance of input sample was indicated as 1,000. **c** The integrity of the RNA collected in each step was assayed by electrophoresis. The input sample was diluted 100 times and the pull-down sample was diluted 10 times. RNA sample (10 μl) was loaded to the electrophoresis. **d** Proteins retained on the beads were analyzed by silver staining. Protein sample (10 μl) was applied to 12 % PAGE and then silver staining

Aiming to identify RNAIII-binding proteins, we performed the pull-down assay using tRSA as a tag. Streptavidin aptamer fusion in the anti-code site of tRNA was captured by streptavidin PMPs, while RNAIII attached to the 3′ site of tRNA can act as a bait for RBPs (Fig. 1A). About 0.3 μg RNA was captured by the SA-PMPs in the pull-down process (Fig. 1B). Although a lot of RNA was degraded, the RNA captured was intact (Fig. 1C), which can act as the bait RNA for protein binding. Proteins retained on the beads were analyzed by silver staining, with proteins binding to empty SA-PMPs as negative controls (Fig. 1D, lane NC), and the major bands were identified by LC/MS (Additional file 1: Table S1). From three biologically independent experiments, 81 proteins were identified and quantified from at least two replicates.

### Classification of the proteins binding to RNAIII

**Fig. 2:**
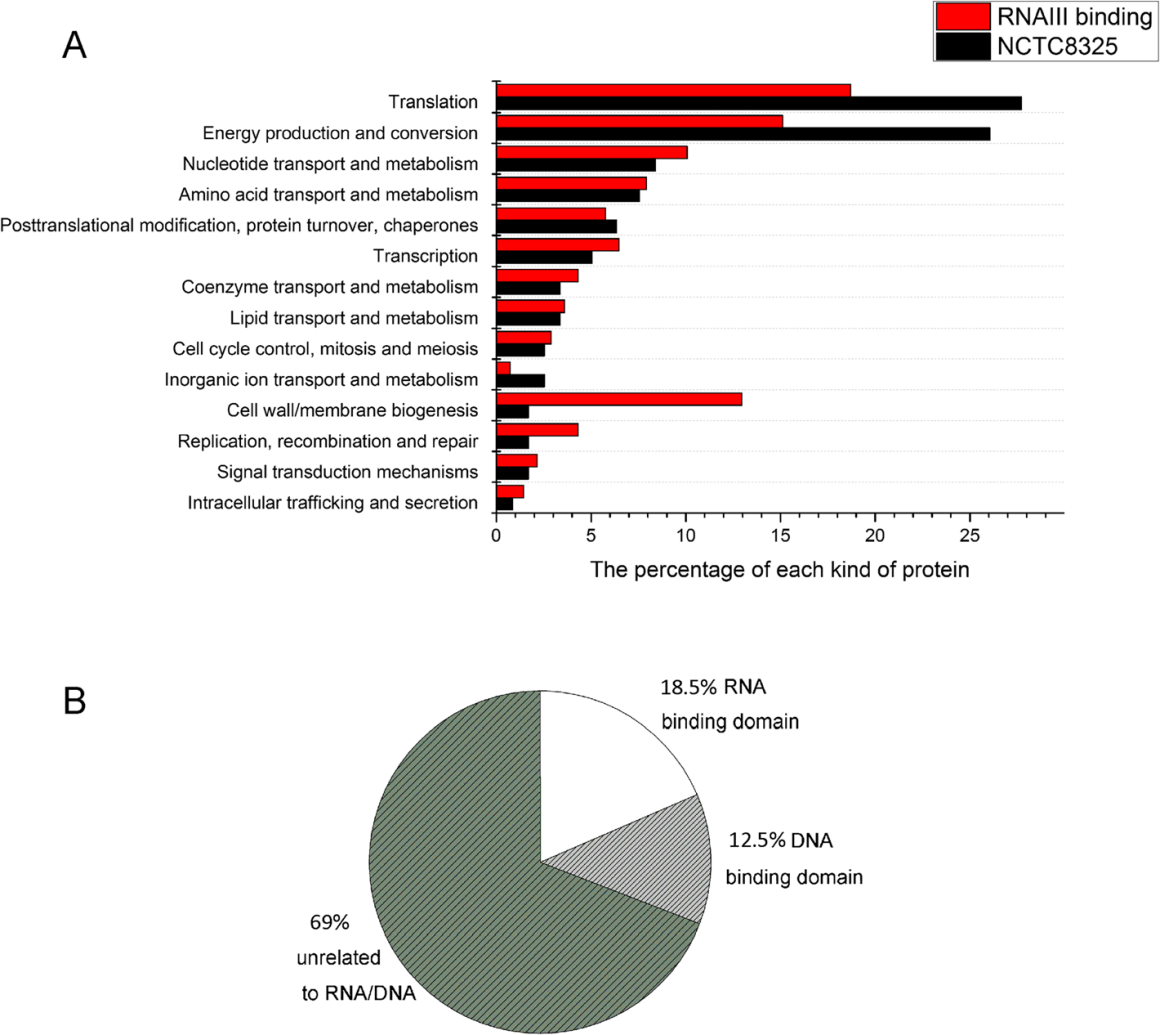
Classification of the proteins binding to RNAIII. **a** The proteins identified by LC-MS were classified based on the COG (Cluster of Orthologous Groups) data in NCBI. The proteins from *S. aureus* strain NCTC8325 was applied to LC-MS and the components of each class of proteins were analyzed the same way. **b** The conserved domains in the identified proteins were generalized based on the Uniprot database

The identified proteins were divided into different classifications based on the COG (Cluster of Orthologous Groups) data in NCBI. The percentage of most kinds of proteins in the pull-down sample was similar to that in the *S. aureus* NCTC8325 proteomics. In contrast, cell wall/membrane biogenesis proteins (15 and 2 in pull down sample and NCTC8325, respectively) and replication-, recombination- and repair-related proteins (5 and 2 in pull-down sample and NCTC8325, respectively) were enriched in the RNAIII pull-down system (Fig. 2A), suggesting that these two kinds of proteins may have higher affinity to RNAIII. These proteins were then classified by the conserved domain analysis. In these 81 proteins, only 18.5 % were predicted to have RNA-binding domains, while 12.5 % contain DNA-binding domains and the others were not known to have relationships with RNA or DNA (Fig. 2B). Those proteins with RNA/DNA-binding motifs were listed in Table 1.

**Table 1:** List of the proteins with RNA-binding and DNA-binding domains identified by MS

Proteins with RNA-binding domains	
Gene	Protein
SAOUHSC_00769	Protein translocase subunit SecA
SAOUHSC_01035	Ribonuclease J 1
SAOUHSC_01252	Ribonuclease J 2
SAOUHSC_01659	Putative uncharacterized protein
SAOUHSC_01679	Putative uncharacterized protein
SAOUHSC_01207	Signal recognition particle protein
SAOUHSC_01184	Sun protein
SAOUHSC_02362	Transcription termination factor Rho
SAOUHSC_01492	GTP-binding protein EngA
SAOUHSC_01163	Pseudouridine synthase
SAOUHSC_01668	GTP-binding protein Era
SAOUHSC_00464	Ribosomal RNA small subunit methyltransferase A
SAOUHSC_01203	Ribonuclease III
SAOUHSC_00513	Putative uncharacterized protein
SAOUHSC_02303	mRNA interferase MazF
Proteins with DNA-binding domains	
Gene	Protein
SAOUHSC_01099	Endonuclease MutS2
SAOUHSC_01351	DNA topoisomerase 4 subunit B
SAOUHSC_00001	Chromosomal replication initiator protein DnaA
SAOUHSC_01682	Chaperone protein DnaJ
SAOUHSC_01850	Catabolite control protein A
SAOUHSC_01576	Exonuclease family protein
SAOUHSC_01454	Putative uncharacterized protein
SAOUHSC_00467	Pur operon repressor
SAOUHSC_00020	Transcriptional regulatory protein WalR
SAOUHSC_01490	DNA-binding protein Hu

### Confirmation of the binding ability of the protein candidates

**Fig. 3:**
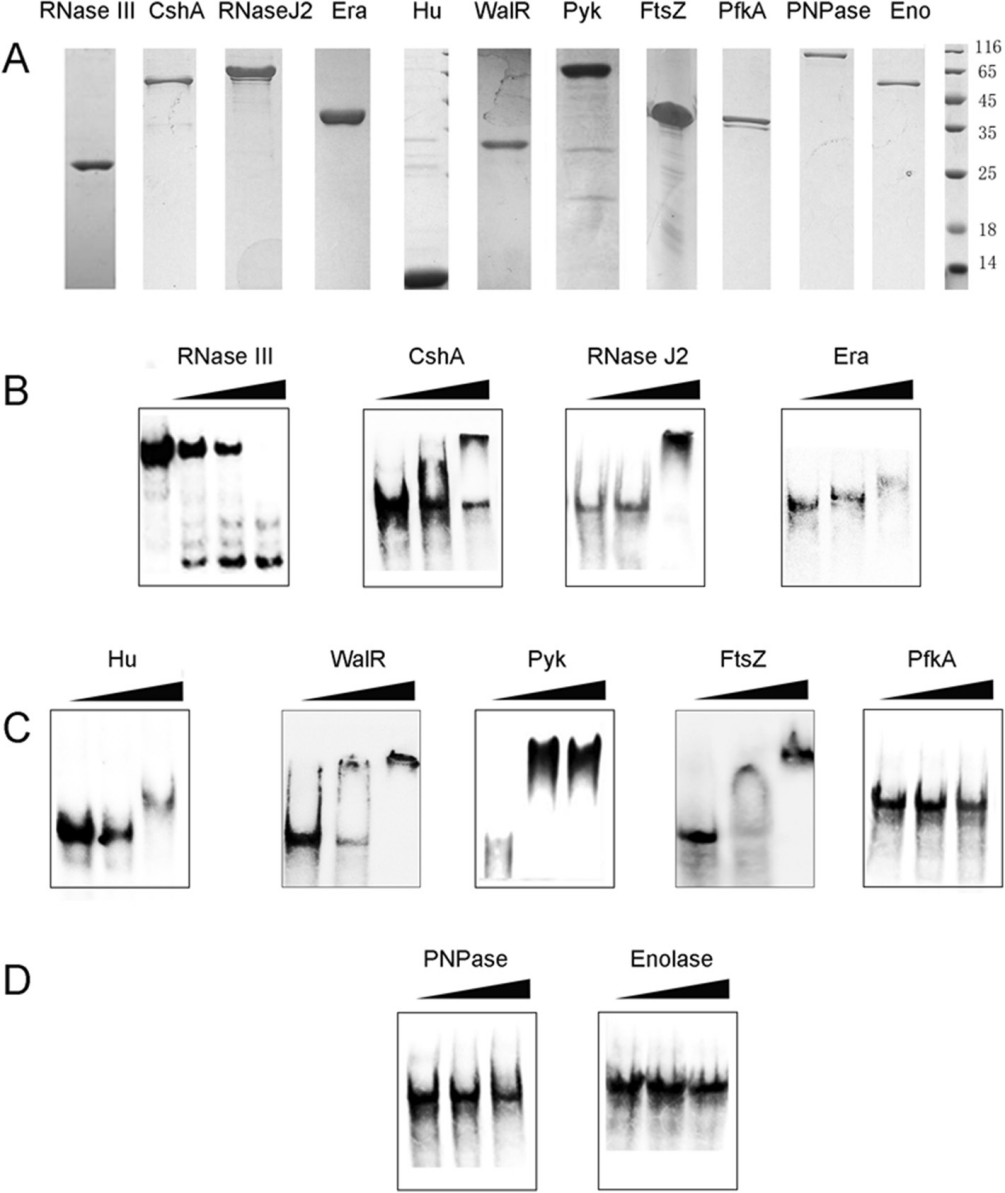
Expression and EMSA of the binding ability of the protein candidates. **a** Protein candidates expressed in *E. coli*. The clone and expression details are listed in Tables 2, 3 and 4. **b**, **c**, and **d** The binding ability of proteins to RNAIII was determined by EMSA. DIG-labeled RNAIII probes (0.24 μM) were used in all reactions. Increasing amounts of different proteins were incubated with labeled RNAIII probes. Figure (**b**) represents four proteins containing RNA-binding motifs (RNase III 0, 2, 4, 8 μM; CshA 0, 6.5, 32 μM; RNase J2 0, 3.2, 6.4 μM; Era 0, 2.9, 5.8 μM.), Figure (**c**) represents two proteins containing DNA-binding motifs, and three proteins unrelated to nucleic acid (Hu 0, 180, 360 μM; WalR 0, 75, 150 μM; Pyk 0, 6.3, 13 μM; FtsZ 0, 27, 140 nM; pfkA 0, 16, 140 μM). Figure (**d**) represents two negative control proteins (PNPase 0, 1.3, 12 μM; Enolase 0, 2.1, 19 μM)

Those protein candidates with RNA or DNA-binding domains have more possibility to interact with RNA because of the structural similarity between RNA and DNA, while the interactions between RNA and other proteins need to be further verified. To confirm that the protein candidates identified were RBPs, the binding ability of these proteins was validated by EMSA. Eleven recombinant proteins with different kind of motifs were expressed in *E. coli*, including four proteins with RNA-binding motifs, two proteins with DNA-binding motifs, and three proteins with no nucleic acid-binding motif. PNPase and Enolase were suggested to be the components of RNA degradosome, but they were not found in our pull-down assay (Fig. 3A).

RNase III has an RNA-binding motif, and exhibited RNA degradation activity (Fig. 3B), which is consistent with the previous report [20]. CshA, RNase J2, and Era also have RNA-binding motifs and showed binding ability with RNAIII (Fig. 3B). Hu and WalR have DNA-binding domain, and also showed RNAIII binding ability (Fig. 3C). Surprisingly, Pyk and FtsZ, without RNA/DNA-binding domains, also had binding activity with RNAIII. PfkA did not bind to RNAIII even at high concentration (Fig. 3C). As expected, PNPase and Enolase did not bind to RNAIII (Fig. 3D), which was consistent with the pull-down assay results. Among the nine proteins identified, eight can interact with RNAIII, suggesting that our pull-down and MS assays are effective and reliable.

## Discussion

The significance of sRNA in gene regulation in bacteria is now widely recognized [1, 21], and more bacterial sRNAs have been identified in the last decade [22, 23]. In *S. aureus*, hundreds of sRNAs have been identified, and some of them have been proved to play roles in physiology and virulence control [24]. The sRNA-mRNA base-pairing mechanism has been found ubiquitously in sRNA regulation in *S. aureus,* whereas, there is currently very limited information about staphylococcal RBPs that might be involved in sRNA regulation [1]. RNase III [20], Hfq [8, 25], and the transcriptional regulator SarA [26] are those only RBPs known in *S. arueus*. RBPs involved in sRNA regulation may include RNA chaperones, RNA helicases, ribonucleases, and some regulators [8]. Also, the activity of some enzymes may be affected by RNA binding [27]. Identification of RBPs in *S. arueus* in this study may help us better understand the details about sRNA regulation in staphylococci and other bacterial pothogens.

tRSA is an efficient RNA aptamer tag for RNA pull-down assay [19, 28, 29]. After invented, tRSA has already been used in different kinds of researches, including microRNA-RNA interaction [28], co-expression of RNA/protein complex, and isolation of RBPs [30]. This RNA aptamer does not need recombinant proteins or affinity matrices compared to those MS2 aptamers; and the labeling is easier and cheaper than biotin. Because there are some standardized reagents for this tag, the effect and stability of the experiment system may be improved a lot. In this study, we used the streptavidin magnetic beads to capture the tRSA tag. Using this system, we have successfully identified proteins binding to RNAIII in *S. aureus*, and this technique may be well suitable for the identification of RBPs in other bacteria.

The proteins captured by RNAIII in our study include various kinds of proteins, some of which have been reported to participate in RNAIII function. RNase III can degrade double-stranded RNA formed by base-pairing of RNAIII with its mRNA targets [1], and it also can digest RNAIII [20]. Era was known to consist of a K homology domain and to recognize the sequence of 16S rRNA of *S. aureus* [31], and we show here it can also bind to RNAIII in vitro. It was reported that eight proteins (CshA, RNase J1, RNase J2, RNase Y, PNPase, Enolase, PfkA and RnpA) may interact with *S. aureus* RNA degradosome, which has been confirmed by bacterial two-hybrid analysis [32]. We here show that CshA and RNase J2 can bind to RNAIII, while PNPase and Enolase cannot. CshA was reported to be involved in mRNA half-life control in *S. aureus* [33], and it may also influence the stability of RNAIII. Besides, we have identified some DNA-binding proteins that can bind to RNAIII, including Hu, which has already been proved to bind to rRNA, tRNA segments, and a few small RNAs related to nucleoid morphology in *E. coli* [34]; WalR is a transcriptional factor involved in autolysis, biofilm formation, and cell wall metabolism. Our results show that WalR can bind to RNAIII as well, which may have the same property as the transcriptional factor SarA [26]. We further show that some metabolism-related proteins can bind to RNAIII, including pyruvate kinase and FtsZ, and this should provide us a new perspective about sRNA-mediated metabolism control.

Another concern in the interaction between RNA and RBPs is the binding specificity. RBPs may recognize specific sequences or structures in their RNA targets [35]. Some of the proteins we identified may bind not only to RNAIII, but also to other RNA or DNA. Further study may illustrate the RNA binding specificity of these RBPs and the relating molecular mechanisms.

## Conclusions

Pull-down assay using tRNA scaffold streptavidin aptamer was performed to search for the proteins binding to RNAIII in *S. aureus*. By using this method, 81 proteins binding to RNAIII were identified by the pull-down and MS assay, and the binding ability of 9 proteins with RNAIII was verified by EMSA. Proteins CshA, RNase J2, Era, Hu, WalR, Pyk and FtsZ were confirmed to bind to RNAIII. The finding and investigation of proteins binding to RNAIII should facilitate sRNA study in staphylococci.

## Methods

### Strains and plasmids

Strains and plasmids used in this study are listed in Table 2. *E. coli* strains were grown at 37 °C in Luria-Bertani (LB) medium with suitable antibiotics: ampicillin (100 μg/mL) or kanamycin (50 μg/mL). *S. aureus* strains were grown at 37 °C in tryptic soy broth (TSB, Oxiod). The media were solidified with 1.5 % (wt/vol) agar as needed.

**Table 2:** Bacteria strains and plasmids used in this study

Strain or plasmid	Comments	Source or Reference
Strains		
*S. aureus* NCTC8325	Wild type	NARSA^a^
*E. coli* DH5α	Clone host strain	Laboratory stock
*E. coli* BL21 (DE3)	Expression host strain	Laboratory stock
*E. coli* M15 (pREP4)	Expression host strain	Laboratory stock
Plasmids		
pcDNA3-tRSA	Template for tRSA	[19]
pEASY TB	Clone vector, Kan^r^ Ap^r^	Transgen
pSXZ06	pEASY TB with *RNAIII* for in vitro transcription	[36]
pET28a (+)	Expression vector with hexahistidine tag, Kan^r^	Novagen
pET21b (+)	Expression vector with hexahistidine tag, Ap^r^	Novagen
pQE30	Expression vector with hexahistidine tag, Ap^r^	QIAGEN
pQE30-RNase III	Expression vector for protein RNase III	[36]
pET28a-Hu	Expression vector for protein Hu	This study
pET21b-FtsZ	Expression vector for protein FtsZ	This study
pET28a-Pyk	Expression vector for protein Pyk	This study
pET28a-Era	Expression vector for protein Era	This study
pET28a-WalR	Expression vector for protein WalR	[37]
pET22b-RNase J2	Expression vector for protein RNase J2	This study
pET22b-CshA	Expression vector for protein CshA	This study
pET22b-PNPase	Expression vector for protein PNPase	This study
pET22b-PfkA	Expression vector for protein PfkA	This study
pET24a-Enolase	Expression vector for protein Enolase	This study

^a^ NARSA, Network on Antimicrobial Resistance in *Staphylococcus aureus*

### In vitro RNA synthesis

The template for tRSA-RNAIII transcription was digested from plasmid pSXZ06. The RNAs were produced by in vitro transcription as described [36], using a RiboMAX Large Scale RNA Production Systems-T7 (Promega). RNAs were labeled with DIG using RNA labeling Mix (Roche).

### RNA isolation and real-time RT-PCR

RNA was extracted using the Trizol method (Invitrogen), and residual DNA was digested with 10 U of DNaseI (Takara) at 37 °C for 1 h. Reverse transcription was carried out with the PrimeScript 1st Strand cDNA synthesis kit (Takara) and real-time PCR was performed with SYBR Premix Ex Taq (TaKaRa) using a StepOne real-time system (Applied Biosystems). The RNA abundance of input sample was indicated as 1,000 and the relevant quantification of captured RNA was analyzed by calculating the difference of CT in the real-time PCR.

### RBPs pull-down assay

RBPs pull-down assay was performed as previously described [19], with some modifications. Briefly, *S. aureus* cultures (2 ml) were collected and bacterial cells were resuspended with lysis buffer (10 mM HEPES, pH 7.0, 200 mM NaCl, 1 % Triton X-100, 10 mM MgCl_2_, 1 mM DTT) containing protease inhibitor cocktail (Sangon), and 40 U/ml lysostaphin (AMBI). The suspension was incubated at 37 °C for 10 min to lyse the cell. After centrifugation at 12,000 g for 30 min, the supernatants were collected and the concentration of total protein was quantified by BCA assay. Avidin (Calbiochem, 10 mg/mg protein), Yeast RNA (Sigma, 0.5 mg/mg protein), and RNase inhibitor (3 μl, Transgen) were added as the final pre-cleared lysates. About 2 mg protein was applied for each pull-down assay.

At the same time, 10 μg of bait RNA sample was dissolved in buffer containing 10 mM HEPES, pH 7.0, 10 mM MgCl_2_, and denatured at 65 °C for 5 min and then cooled to room temperature. SA-PMPs (0.6 mg, Promega) was rinsed twice with 0.5 × SSC, and then rinsed twice with lysis buffer. The RNA sample was incubated with the prepared SA-PMPs at 4 °C for 20 min on a rotating shaker, then pre-cleared lysates were added and incubated for another 1.5 h on a rotating shaker. The SA-PMPs was then washed 3 times with fresh lysis buffer. SDS-loading buffer (50 μl) was added and the proteins captured were incubated at 95 °C for 10 min. Samples from each step were collected for RNA assay.

The proteins were separated by SDS-PAGE and silver or Coomassie brilliant blue staining, and the gel bands were excised and in-gel digested with trypsin (0.6 mg), and the tryptic peptides were subjected to LC-MS/MS analysis with a LTQ mass spectrometer (ProteomeX-LTQ, ThermoFisher Scientific). Sequence and peptide fingerprint data were analyzed using the NCBI database.

### Expression and purification of recombinant proteins

Open reading frames of different proteins were amplified from *S. aureus* NCTC8325 genomic DNA with primers listed in Table 3. After digested, the PCR product was ligated into plasmid, then transformed into *E. coli* DH5α, and then the expression strains. The plasmids, expression strains, and conditions for proteins were listed in Tables 2 and 4. The recombinant proteins were purified by Ni-NTA resin (Qiagen), eluted, passed through an ultrafiltration system to remove imidazole, and then stored in 10 % glycerol at -70 °C until use. Protein purity and concentration were determined by SDS-PAGE and the BCA assay.

**Table 3:** Primers used in this study

Primer name	Sequence
tRSA-f	GCCGCCAGTGTGCTGGAATT
tRSA-r-EcoRI-BamHI	GCGGGATCCGATATCTGCAGAATTC
Vitro-RNAIII-f-EcoRI	GCG GAATTC AGATCACAGAGATGTGAT
Vitro-RNAIII-r-BamHI	GCG GGATCC AAGGCCGCGAGCTTGGGA
RNase III-f-BamHI	TAAAGGATCTCTAAACAAAAGAAAAGTGAGATAGTTAATC
RNase III-r-SmaI	TACCCCCGGGTTTAATTTGTTTTAATTGCTTATAGGCACTTTTAG
Hu-r-XhoI	CCG CTCGAG TTTTACAGCATCTTTTA
Hu-f-NcoI	CATG CCATGG GC AACAAAACAGATTT
FtsZ-f-NdeI	CGCCATATGTTAGAATTTGAAC
FtsZ-r-XhoI	CCGCTCGAGACGTCTTGTTCTTC
Era-f-Nde I	CCCATATGACAGAACATAAATCAGG
Era-r-Xho I	CCGCTCGAGTTAATCTTGGTCTTCAAC
Pyk-f-BamHI	CGCGGATCCATGAGAAAAACTAAAATTGT
Pyk-r-xhoI	GTTCTCGAGTTATAGTACGTTTGCATATCCTTC
WalR-f-NcoI	CCACCATGGCTAGAAAAGTTGTTGTAGTTG
WalR-r-XhoI	CTCCTCGAGCTCATGTTGTTGGAGGAAATATCCA
RNase J2-f-BamHI	CGCGGATCCATGAATAGTGAGTTTATATATGGACGGGTAACAAATTTAGG
RNase J2-r-XhoI	CCGCTCGAGTTAAATTTCAGAAATTACTGGAATAATCATAGGACGACG
CshA-f-BamHI	GGATCCATGCAAAATTTTAAAGAACTAGGGATTTCG
CshA-r-XhoI	CTCGAGTTTTTGATGGTCAGCAAATGTGCG
PNPase-f-BamHI	GAAGGATCCATGTCTCAAGAAAAGAAAGTTTTTAAAAC
PNPase-r-XhoI	GAACTCGAGTTCTTCTAATGCTCTGTGTGATGC
PfkA-f-BamHI	GCCGGATCCATGAAGAAAATTGCAGTTTTAACTAGTGGT
PfkA-r-XhoI	CCCCTCGAGTATAGATAACTTGTTAGCAAGTTCATAT
Enolase-f-BamHI	GAAGGATCCATGCCAATTATTACAGATGTTTACGC
Enolase-r-XhoI	GAACTCGAGTTTATCTAAGTTATAGAATGATTTGATACCG

**Table 4:** Conditions used for protein expression

Name	OD_600_	IPTG	Temperature	Buffer
RNase III	0.2	1 mM	30 °C for 4 h	30 mM Tris–HCl, 500 mM KCl, 0.1 mM DTT, 0.1 mM EDTA, pH 8.0
Hu	0.8	1 mM	16 °C for 16 h	50 mM PBS, 150 mM NaCl, pH 7.8
FtsZ	0.6	0.5 mM	20 °C for 16 h	50 mM Tris–HCl, 300 mM NaCl, pH 8.0
Pyk	0.6	0.2 mM	30 °C for 5 h	30 mM Tris–HCl, 200 mM NaCl, pH 7.8
Era	0.6	0.5 mM	16 °C for 16 h	50 mM Tris–HCl, 500 mM NaCl, pH 8.0
WalR	0.6	0.5 mM	20 °C for 16 h	50 mM Tris–HCl, 200 mM NaCl, pH 8.0
RNase J2	0.6	0.4 mM	16 °C for 20 h	20 mM Tris–HCl, 200 mM NaCl, pH 7.5
CshA	0.6	0.4 mM	16 °C for 20 h	20 mM Tris–HCl, 1 M NaCl, pH 7.5
PNPase	0.6	0.4 mM	16 °C for 20 h	20 mM Tris–HCl, 200 mM NaCl, pH 7.5
PfkA	0.6	0.4 mM	16 °C for 20 h	20 mM Tris–HCl, 200 mM NaCl, pH 7.5
Enolase	0.6	0.4 mM	16 °C for 20 h	20 mM Tris–HCl, 200 mM NaCl, pH 7.5

### EMSA

EMSA between RNA and RBPs was performed as previously described [34]. The labeled RNA was denatured at 65 °C for 10 min and cooled to room temperature. Increasing amounts of proteins were incubated with RNA on ice for 30 min in protein buffer. After electrophoresis in 4 % native polyacrylamide gel in a 1 × TBE buffer, RNA was electrotransferred to a charged nylon membrane (Millipore) in 0.5 × TBE. The DIG-labeled RNA was detected using a DIG gel-shift kit (Roche) according to the manufacturer’s instructions.

## Additional file

Additional file 1: Table S1.
**List of proteins identified by MS.**

## Abbreviations

sRNA: Small RNA
*S. aureus*: *Staphylococcus aureus*
RBPs: RNA-binding proteins
tRSA: tRNA scaffold to a streptavidin aptamer
SA-PMPs: Streptavidin MagneSphere Paramagnetic Particles
MS: Mass spectrometry
EMSA: Electrophoresis mobility shift assay
